# Comparative transcriptome analysis of synthetic and common wheat in response to salt stress

**DOI:** 10.1038/s41598-022-15733-2

**Published:** 2022-07-07

**Authors:** Rio Nakayama, Mohammad Taheb Safi, Waisuddin Ahmadzai, Kazuhiro Sato, Kanako Kawaura

**Affiliations:** 1 Kihara Institute for Biological Research, Yokohama City University, Yokohama, 244-0813 Japan; 2 Institute of Plant Science and Resources, Okayama University, Kurashiki, 710-0046 Japan

**Keywords:** Plant breeding, Salt

## Abstract

Salt stress reduces wheat yield. Therefore, improvement for enhanced salt stress tolerance is necessary for stable production. To understand the molecular mechanism of salt tolerance in common wheat and synthetic hexaploid (SH) wheat, RNA sequencing was performed on the roots of three wheat lines salt-tolerant SH wheat, salt-tolerant common wheat, and salt-sensitive common wheat. Differentially expressed genes (DEGs) in response to salt stress were characterized using gene ontology enrichment analysis. Salt tolerance in common wheat has been suggested to be mainly regulated by the activation of transporters. In contrast, salt tolerance in SH wheat is enhanced through up-regulation of the reactive oxygen species signaling pathway, other unknown pathways, and different ERF transcription factors. These results indicate that salt tolerance is differentially controlled between common wheat and SH wheat. Furthermore, QTL analysis was performed using the F_2_ population derived from SH and salt-sensitive wheat. No statistically significant QTL was detected, suggesting that numerous QTLs with negligible contributions are involved in salt tolerance in SH wheat. We also identified DEGs specific to each line near one probable QTL. These findings show that SH wheat possesses salt tolerance mechanisms lacking in common wheat and may be potential breeding material for salt tolerance.

## Introduction

Salt stress is a major environmental stress that reduces crop yield. Plants are constantly exposed to environmental stresses because of their inability to move and escape from undesirable environments. Salt stress caused by soil salinity leads to serious damage to plant growth and development. Salinity induces osmotic stress and ionic toxicity, which affect plant growth^1^. In the early phase, sodium ions induce osmotic stress, which reduces water availability and cell turgor pressure, inhibiting young leaf growth. After accumulating sodium ions in the shoot, ionic toxicity affects metabolism and inhibits photosynthesis.

Salt tolerance mechanisms in plants have been studied at the molecular genetic level^2,3^. When roots are exposed to salt stress, in the early phase, sodium ions enter the cell through non-selective cation channels and induce calcium waves and reactive oxygen species (ROS) signaling. Calcium ion signals activate kinases, such as calcium-dependent protein kinases. Calcineurin B-like proteins (CBLs) bind calcium and activate CBL-interacting protein kinases (CIPKs) by forming CBL–CIPK complexes. ROS are generated by respiratory burst oxidase homologs (RBOHs), which are NADPH oxidases. These signals regulate salt-responsive genes, such as transcription factors, ion channels, and transporters by changing the biosynthesis of phytohormones. The salt overly sensitive (SOS) pathway is a well-characterized pathway. When salt stress induces calcium waves, calcium is sensed by SOS3, the CBL protein^4^. SOS3 interacts with SOS2, which is a CIPK and forms the SOS2–SOS3 kinase complex^5^. The SOS2–SOS3 kinase complex phosphorylates SOS1, a Na^+^/H^+^ antiporter (NHX)^6^. Activated SOS1 localized at the plasma membrane exports Na^+^ from the cell and maintains low Na^+^ in the cytoplasm.

Wheat is a staple crop that is cultivated worldwide. However, wheat is less tolerant to salinity than barley, and in general, monocots are less salt-tolerant than dicots^7^. Common wheat (*Triticum aestivum* L., AABBDD genome) evolved through natural hybridization between a cultivated emmer wheat (*T. turgidum* L., AABB genome) and wild goat grass *(Aegilops tauschii* Coss., DD genome) about 8000 years ago^8^. The common wheat D genome exhibits low genetic diversity. Therefore, synthetic hexaploid (SH) wheat has been systematically developed by artificially crossing tetraploid wheat with *Ae. tauschii* to utilize genetic diversity in breeding programs^9^. Some SH wheat lines showed higher salt tolerance than their parental tetraploid lines, and synthetic lines produced by crossing salt-sensitive tetraploid lines with salt-tolerant diploid lines showed higher salt tolerance than salt-tolerant cultivars^10^. Therefore, synthetic wheat has the potential to adapt to environmental stress that is lacking in common wheat.

Wheat has a large complex genome; however, precise chromosome level genome assemblies have been recently released in several accessions^11,12^. Additionally, RNA sequencing (RNA-seq) has become a convenient and indispensable tool to analyze differential gene expression^13^. This enables us to perform a genome-scale analysis, even in wheat. Transcriptome analyses have been conducted to understand the salinity tolerance molecular mechanisms in wheat^14–16^. They have revealed that the expression of numerous genes, including SOS genes, transcription factors, and kinases, is altered in response to salt stress in common wheat, as in other plants.

In the present study, we performed transcriptome analysis to characterize early salt stress response in the roots of SH wheat, which showed high salt tolerance, and compare it to the transcriptome of common wheat. Furthermore, we aimed to clarify the genetic factors related to the salt stress response of synthetic wheat by performing quantitative trait locus (QTL) analysis using the F_2_ population from a crossing a salt tolerant SH wheat and a landrace. We found that SH wheat responds early to salt stress by activating genes in signaling pathways different from those in common wheat.

## Results

### Evaluation of salt tolerance in synthetic wheat and common wheat

**Figure 1:**
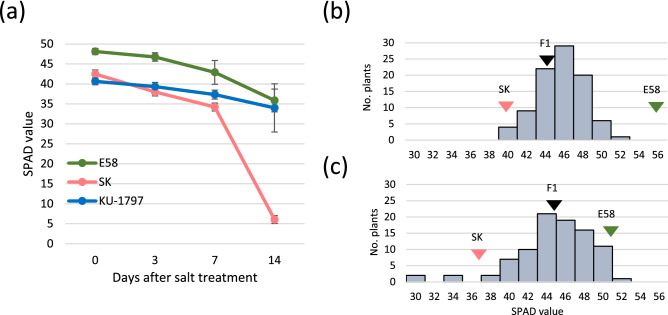
SPAD value changes in response to salt stress. (**a**) SPAD value of leaf after 150 mM NaCl treatment. (**b**) Distribution of the frequencies of SPAD value after 7 d of NaCl treatment in the F_2_ population. (**c**) Distribution of the frequencies of SPAD value after 14 d of NaCl treatment in the F_2_ population.

We analyzed three genotypes: cultivar Shirasagi-komugi (KT020-019; SK), accessions of KU-1797 and Elite#1–58 (E58). The plants were treated with 150 mM NaCl to induce salt stress, and SPAD values indicating chlorophyll content were measured at 3, 7, and 14 d after NaCl treatment (Figs. 1, S1). E58 and KU-1797 showed high SPAD values before NaCl treatment and showed significantly higher SPAD values than SK at 14 days after NaCl treatment. These results suggested that both E58 (SH wheat) and KU-1797 (common wheat) were more tolerant to salinity than SK.

### RNA-seq reads were mapped on the reference genome of common wheat and differentially expressed genes (DEGs) were selected

RNA-seq was performed to compare the early response to salt stress among three lines. We obtained 3.17–13.3 Gb of sequences in each sample, which were subjected to quality control using FASTQC and 92.2–96.3% of the sequences showed quality of more than Q30 (Supplementary Table S1). Reads were trimmed using trimmomatic and mapped onto IWGSC RefSeq v. 1.0^11^. The ratio of reads uniquely aligned to the genome was 67.9–83.6%. The three lines had similar alignment ratios. Therefore, DEGs were selected for further analysis using the alignments.

**Figure 2:**
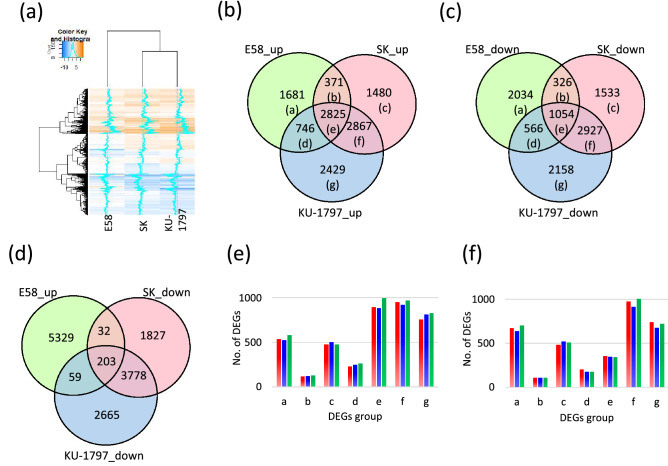
Differentially expressed genes (DEGs) in response to salt stress in three wheat lines. (**a**) Heat map and hierarchical clustering of DEGs. (**b**) Venn diagram of up-regulated DEGs (**c**) Venn diagram of down-regulated DEGs. Parenthesized alphabet shows a group in (**e**), (**f**), and Table 1. (**d**) Venn diagram of up-regulated DEGs in E58 and down-regulated DEGs in SK and KU-1797. (**e**) The number of up-regulated DEGs represented in (**b**) mapped to each sub-genome. (**f**) The number of down-regulated DEGs represented in (**c**) mapped to each sub-genome. Red, blue and green bars indicate A, B, and D genomes, respectively.

DEGs were determined by comparing the read counts between the control and salt-treated samples in each line. Among DEGs that significantly changed the read counts by more than two-fold (P < 0.01), 5623, 7543, and 8867 genes were up-regulated by NaCl treatment, and 3980, 5840, and 6705 genes were down-regulated in E58, SK, and KU-1797, respectively. The total number of DEGs was 9603, 13,383, and 15,572 in E58, SK, and KU-1797, respectively, indicating that fewer genes responded to salt stress in the SH wheat than in other two common wheat lines. To characterize the overall response to salt in each line, hierarchical clustering was performed using log_2_ converted fold change value (Log2FC) of DEGs (Fig. 2). The expression patterns of common wheat lines, namely SK and KU-1797, were grouped, and that of SH wheat, E58, was distinguished. These results imply that salt-responsive genes in SH wheat are different from those in common wheat.

### Characterization of salt-responsive genes in each line

DEGs were separated into up-regulated and down-regulated genes. They were compared with three lines using a Venn diagram (Fig. 2). Among the DEGs, 1681 up-regulated and 2034 down-regulated genes were specific to E58. In contrast, 2825 and 1054 genes were up-regulated and down-regulated in all three lines, respectively. The number of DEGs shared between the two common wheat lines was greater than that between common and SH wheat lines, suggesting that the response to salt stress was different between common and SH wheat.

To examine whether a specific genome contributes to the salt-stress response, DEGs were grouped according to the Venn diagram, and the number of genes located in each genome was counted (Fig. 2; Table 1). Among the up-regulated genes, those mapped to the D genome tended to be more than those to A or B genome, except for genes specifically up-regulated in SK (group c). Among the down-regulated genes, those mapped to the D genome of E58 were a little more than other genomes, while a clear difference was not observed among the groups (Fig. 2). In addition, we mapped all the DEGs to the reference genome to estimate their positions (Figs. S2, S3 and S4). Their Log2FC values were plotted and compared between the SH and common wheat lines. DEGs in both SH and common wheat lines were mapped to the entire genome, and no bias in position on the chromosome was observed in this resolution.

**Table 1:** DEGs mapped to each genome.

Group in the Venn diagram	Description of expression	A genome		B genome		D genome		Unknown		Total no. of DEGs
		No DEGs	(%)	No DEGs	(%)	No DEGs	(%)	No DEGs	(%)	
**Up-regulated**										
a	Only E58	538	32.0	524	31.2	583	34.7	36	2.1	1681
b	E58, SK	118	31.8	119	32.1	127	34.2	7	1.9	371
c	Only SK	477	32.2	503	34.0	477	32.2	23	1.6	1480
d	E58, KU-1797	231	31.0	247	33.1	262	35.1	6	0.8	746
e	All	895	31.7	884	31.3	997	35.3	49	1.7	2825
f	SK, KU-1797	953	33.2	920	32.1	968	33.8	26	0.9	2867
g	Only KU-1797	758	31.2	810	33.3	830	34.2	31	1.3	2429
	Total	3970	32.0	4007	32.3	4244	34.2	178	1.4	12,399
**Down-regulated**										
a	Only E58	669	32.9	635	31.2	700	34.4	30	1.5	2034
b	E58, SK	106	32.5	109	33.4	106	32.5	5	1.5	326
c	Only SK	481	31.4	522	34.1	509	33.2	21	1.4	1533
d	E58, KU-1797	204	36.0	177	31.3	176	31.1	9	1.6	566
e	All	354	33.6	348	33.0	340	32.3	12	1.1	1054
f	SK, KU-1797	975	33.3	913	31.2	1003	34.3	36	1.2	2927
g	Only KU-1797	740	34.3	677	31.4	719	33.3	22	1.0	2158
	Total	3529	33.3	3381	31.9	3553	33.5	135	1.3	10,598

In addition to the line-specific DEGs, we found DEGs that showed opposite expression patterns between SH wheat and common wheat; 203 DEGs were up-regulated in SH wheat and down-regulated in two common wheat lines, and 43 DEGs were down-regulated in SH wheat and up-regulated in two common wheat lines (Fig. 2). Among the 203 specifically up-regulated genes in E58, 66, 57, 79, and 1 genes were mapped to A, B, D genomes, and unknown position, respectively, while 15, 9, 18, and 1 of the 43 down-regulated genes in E58 were mapped to A, B, D genomes and unknown position, respectively. A higher proportion of the DEGs was observed on the D genome. These genes derived from the D genome of *Ae. taucshii* might have an important function in salt tolerance in SH wheat.

### Gene ontology (GO) analysis showed that the function of salt-responsive genes differed between the synthetic and common wheat

**Figure 3:**
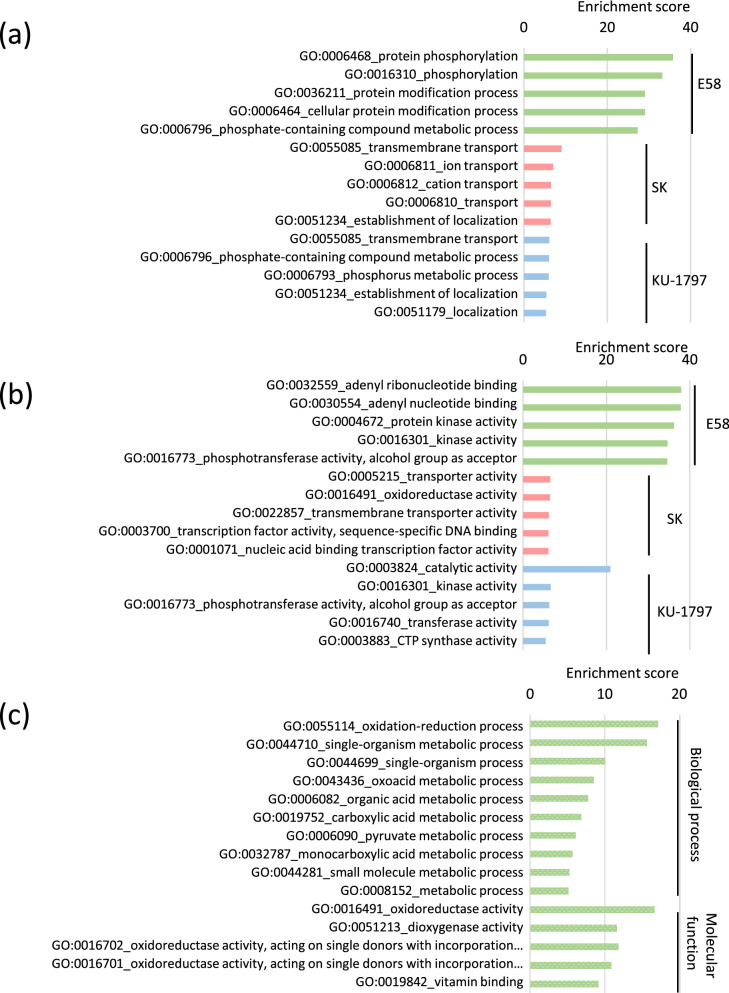
GO enrichment analysis for specific DEGs in each line. (**a**) Biological process enriched in each line-specific up-regulated DEGs (**b**) Molecular function enriched in each line-specific up-regulated DEGs (**c**) Biological process and molecular function enriched in DEGs which were up-regulated in E58 and down-regulated in both SK and KU-1797.

GO enrichment analysis was performed to estimate the function of specific DEGs in each line. The GO terms were divided into the biological process (BP), molecular function (MF), and cellular component (CC), and the top five GO terms of BP and MF with high enrichment scores are shown in Fig. 3. Among the up-regulated genes, the GO term classified in BP with the highest enrichment score was “protein phosphorylation” in SH wheat, whereas it was “transmembrane transport” in SK and KU-1797. The enrichment scores were 35.7, 9.0, and 6.1 in E58, SK, and KU-1797, respectively, showing higher scores in SH wheat. Among the down-regulated genes, the GO term of the “single-organism process” was significantly higher in SH wheat, followed by the “oxidation–reduction process” (Fig. S5). The GO terms of “DNA metabolic process” and “replication” were enriched in SK and KU-1797, respectively. Among the up-regulated genes, the GO terms classified into MF with significantly high enrichment scores were “adenyl ribonucleotide binding”, “transporter activity”, and “catalytic activity” in E58, SK, and KU-1797, respectively (Fig. 3). Among the down-regulated genes, the GO term of “oxidoreductase activity” was significantly enriched, followed by “nucleic acid binding transcription factor activity” in E58 (Fig. S5). The GO terms of “catalytic activity”, “hydrolase activity”, and” acting on glycosyl bonds” were highly enriched in SK and KU-1797. Among the GO terms classified into CC, the up-regulated DEGs in SK and KU-1797, and down-regulated DEGs in E58 were enriched in the GO terms related to cellular membranes (Fig. S5). These results suggested that although the level of salt tolerance in the two common wheat lines was different, they showed similar responses to salt stress. In contrast, SH wheat showed different responses to common wheat. In addition, predominant genes induced in response to salt stress in SH wheat were related to signal transduction.

We further performed GO enrichment analysis to estimate the function of DEGs that showed opposite expression patterns between SH wheat and two common wheat lines (Fig. 2). Among these DEGs up-regulated in SH wheat, the GO term as the “oxidation-reduction process” was the most enriched, followed by the “single-organism metabolic process” of the GO terms classified into BP (Fig. 3). Similarly, GO terms of MF, namely “oxidoreductase activity” and “dioxygenase activity”, were significantly enriched (Fig. 3). In addition, the number of GO terms enriched for “cation binding” and “calcium ion binding” was significant, suggesting that ion binding activity was different. Furthermore, 9 DEGs were found to have GO terms for “nucleic acid binding transcription factor activity”, “transcription factor activity”, and “sequence-specific DNA binding”. Analysis of homologous genes in rice showed that all 9 genes showed homology with OsERF transcription factors. These ethylene-responsive factors (ERFs) may have specific functions in response to salt stress in E58.

### Kyoto encyclopedia of gene and genomes (KEGG) pathway analysis showed that the phenylpropanoid biosynthesis pathway was up-regulated in SH wheat

KEGG^18^ pathway enrichment analysis was performed using rice orthologous genes to estimate SH wheat’s characteristic pathways under salt stress. Specific DEGs in each line (groups a, c, and g in Fig. 2) were selected and searched for orthologs in rice. Among the 1681 up-regulated and 2034 down-regulated genes specific to E58 (group a), 1461 and 1926 genes, respectively, were orthologous in rice. Pathway analysis indicated that the phenylpropanoid biosynthesis pathway was up-regulated, and the photosynthesis pathway was down-regulated in E58 (Table 2). Meanwhile, 1391 and 1431 up-regulated genes and 1141 and 1525 down-regulated genes in SK (group c) and KU-1797 (group g), respectively, were orthologous in rice. In both lines, the plant hormone signal transduction pathway was up-regulated, and the phenylpropanoid biosynthesis pathway, which was up-regulated in E58, was down-regulated. These results support the hypothesis that different pathways in response to salt stress are activated or suppressed between SH and common wheat.

**Table 2:** Enriched pathway by KEGG pathway analysis.

Group in the Venn diagram	Line	Term	Description	p-value
**Up- regulated**				
a	E58	osa00940	Phenylpropanoid biosynthesis	2.E−03
a	E58	osa04626	Plant-pathogen interaction	2.E−02
a	E58	osa00350	Tyrosine metabolism	2.E−02
a	E58	osa00100	Steroid biosynthesis	2.E−02
a	E58	osa00500	Starch and sucrose metabolism	3.E−02
a	E58	osa00270	Cysteine and methionine metabolism	3.E−02
c	SK	osa04075	Plant hormone signal transduction	2.E−03
c	SK	osa00591	Linoleic acid metabolism	4.E−03
c	SK	osa00480	Phenylalanine metabolism	2.E−02
c	SK	osa00592	Flavonoid biosynthesis	4.E−02
c	SK	osa04626	Glyoxylate and dicarboxylate metabolism	5.E−02
g	KU-1797	osa04075	Plant hormone signal transduction	4.E−03
g	KU-1797	osa00591	Linoleic acid metabolism	5.E−02
**Down-regulated**				
a	E58	osa00195	Photosynthesis	7.E−06
a	E58	osa00940	Phenylpropanoid biosynthesis	1.E−04
a	E58	osa00630	Glyoxylate and dicarboxylate metabolism	1.E−03
a	E58	osa00480	Glutathione metabolism	9.E−03
a	E58	osa00360	Phenylalanine metabolism	1.E−02
a	E58	osa00903	Limonene and pinene degradation	5.E−02
c	SK	osa00940	Phenylpropanoid biosynthesis	6.E−05
c	SK	osa00360	Phenylalanine metabolism	8.E−04
c	SK	osa00941	Flavonoid biosynthesis	9.E−03
g	KU-1797	osa00940	Phenylpropanoid biosynthesis	5.E−05
g	KU-1797	osa00360	Phenylalanine metabolism	5.E−03
g	KU-1797	osa03030	DNA replication	1.E−02
g	KU-1797	osa00500	Starch and sucrose metabolism	1.E−02
g	KU-1797	osa04075	Plant hormone signal transduction	2.E−02
g	KU-1797	osa00860	Porphyrin and chlorophyll metabolism	3.E−02
g	KU-1797	osa00040	Pentose and glucuronate interconversions	4.E−02
g	KU-1797	osa03410	Base excision repair	4.E−02

### Salt-overly sensitive (SOS) signaling genes were up-regulated mainly in common wheat

We found DEGs that showed differential expression between SH wheat and common wheat. We then focused on salt-tolerant related genes involved in SOS signaling, namely *TaSOS1*, *TaSOS2*, and *TaSOS3*.

**Figure 4:**
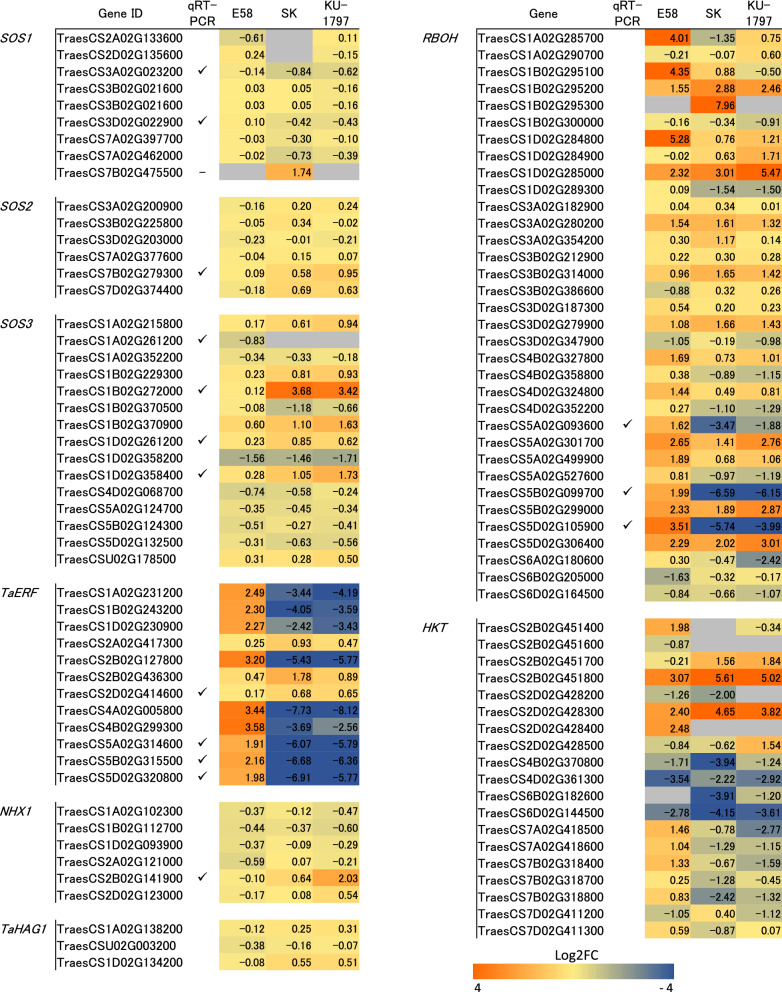
Relative expression level after NaCl treatment revealed by RNA-seq. Color shows the log_2_ converted fold change (Log2FC) of expression.

**Figure 5:**
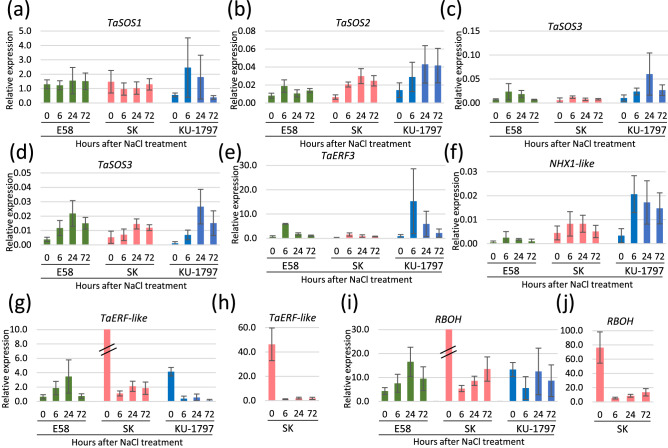
Comparison of salt-responsive gene expression pattern in three lines. (**a**) *TaSOS1* (TraesCS3D02G022900) (**b**) *TaSOS2* (TraesCS7B02G279300) (**c**) *TaSOS3* (TraesCS1D02G358400) (**d**) *TaSOS3* (TraesCS1B02G272000) (**e**) *TaERF3* (TraesCS2D02G414600) (**f**) *NXH1-like* gene (TraesCS2B02G141900) (**g**, **h**) *TaERF-like* gene (TraesCS5D02G320800) (**i**, **j**) *RBOH* (TraesCS5A02G093600) (**h**, **j**) Scale-up of expression level in SK. Expression level relative to *Actin* expression level revealed by qRT-PCR.

In the wheat reference genome, 89 loci for *TaSOS1* have been characterized^19^. Among them, nine *TaSOS1* genes that have high homology with *AtSOS1* and are expressed in more than one sample were chosen, and Log2FC was displayed (Fig. 4). One *TaSOS1* (TraesCS7B02G475500) was up-regulated more than two-fold in SK, as revealed by RNA-seq, but the expression was not detected by qRT-PCR (data not shown). In contrast, three *TaSOS1* genes (TraesCS3A02G023200, TraesCS3B02G021600, TraesCS3D02G022900), which were considered to be homoeologous genes and were not categorized as DEGs (Fig. 4), showed similar expression patterns in E58 and SK but different from KU-1797 (Fig. 5). The expression at 6 and 24 h after NaCl treatment tended to be up-regulated in KU-1797, but the level was almost similar to that of the control (0 h) in E58 and SK (Fig. 5).

Six *TaSOS2* genes with high homology with *AtSOS2* were selected and displayed Log2FC (Fig. 4). No genes were categorized as DEGs displayed Log2FC > 1. The expression patterns of three *TaSOS2* (TraesCS7A02G377600, TraesCS7B02G279300, and TraesCS7D02G374400), which are homoeologous genes, were investigated by qRT-PCR (Fig. 5). *TaSOS2* genes tended to be up-regulated during NaCl treatment in SK and KU-1797 but slightly up-regulated only at 6 h in E58 (Fig. 5), suggesting that *TaSOS2* genes were differentially regulated in response to salt stress between SH wheat and common wheat.

Fifteen *TaSOS3* genes with high homology with *AtSOS3* were differentially expressed in the three lines (Fig. 4). The expression levels of two sets of homoeologous gene groups, one containing two genes (TraesCS1B02G370900 and TraesCS1D02G358400) and another containing three genes (TraesCS1D02G261200, TraesCS1B02G272000, and TraesCS1A02G261200), were quantified using qRT-PCR. Although the former two genes were identified as DEGs in SK and KU-1797 via RNA-seq using 6 h treatment samples, the expression was more induced at 24 h in KU-1797 but not induced in E58 and SK (Fig. 5). The other three genes were not categorized as significant DEGs (Fig. 4). Using qRT-PCR, these genes showed similar expression patterns in the three lines and were most induced after 24 h of NaCl treatment (Fig. 5). The expression levels were higher in E58 and KU-1797 than in SK, suggesting that these *TaSOS3* genes were commonly related to salt response in synthetic wheat and salt-tolerant KU-1797. The SOS pathway was involved in E58 and KU-1797 in response to salt stress, while the expression of these genes was more up-regulated in KU-1797 than in E58.

### Specific *RBOHs* and *ERFs* were up-regulated in SH wheat and down-regulated in common wheat

We focused on reactive oxygen species (ROS) signaling and ERF transcription factors. ROS signaling along with Ca^2+^ has been reported as salt-induced early signaling^3^. We found that genes encoding RBOHs showed opposite expression patterns in SH and common wheat (Figs. 2 and 4). Three homoeologous genes (TraesCS5A02G093600, TraesCS5B02G099700, and TraesCS5D02G105900) were confirmed to have different expression patterns between SH and common wheat via qRT-PCR (Fig. 5). In contrast, it has been suggested that ROS production is mediated by the histone acetyltransferase TaHAG1, which enhances salt tolerance in SH and hexaploid wheat rather than tetraploid wheat^20^. We investigated the Log2FC of *TaHAG1* (TraesCS1A02G138200, TraesCSU02G003200, and TraesCS1D02G134200) and observed no significant differential expression (Fig. 4).

Furthermore, genes coding for the ERF transcription factor showed differential expression between SH and common wheat lines (Fig. 2). We selected three homoeologous genes (TraesCS5A02G314600, TraesCS5B02G315500, and TraesCS5D02G320800) coding ERFs that were named *TaERF-like* genes, which showed opposite expression in SH and common wheat (Fig. 4). The different expression patterns over time were confirmed using qRT-PCR (Fig. 5). A study reported *TaERF3* to be involved in salt and drought tolerance in wheat^21^. Therefore, we examined the expression pattern of *TaERF3* using qRT-PCR. *TaERF3* expression was induced in E58 and KU-1797 but not in SK. These results suggest that *TaERF3* is commonly related to salt-tolerance in both SH and common wheat, and that *RBOH* and *TaERF-like* genes are involved in salt response specifically in E58.

### A Na^+^/H^+^ antiporter was up-regulated in salt-tolerant common wheat

Ion channels, transporters, and antiporters function to exclude or sequester Na^+^ and maintain K^+^ homeostasis, which plays an important role in salt tolerance in plants^3^. The NHX family is important for maintain ion homeostasis at the cellular level^1^. We found that DEG (TraesCS2B02G141900), having homology with *NHX1*, was significantly up-regulated in KU-1797 (Fig. 4). We named this gene *NHX1-like* and investigated its expression pattern over time using qRT-PCR (Fig. 5). The expression of the *NHX1-like* gene was up-regulated at 6, 24, and 72 h after NaCl treatment only in KU-1797 but was not up-regulated over time in E58 (Fig. 5). The other family of high-affinity potassium transporters (HKT) is also important for acquiring salt tolerance in plants^1^. In wheat, *TaHKT1;5-D* moderated Na^+^ accumulation in shoots and was reported as a candidate gene for the salt-tolerant locus, *Kna1*^22^. It has been suggested that up-regulation of *TaHKT1;5* contributes to salt tolerance through allopolyplodization^23^. We searched 19 genes having homology with *TaHKT1;5-D* and displayed values of log2FC (Fig. 4). *TaHKT1:5-D* (TraesCS4D02G361300) and its homoeologous gene *TaHKT1;5-B* (TraesCS4B02G370800) were down-regulated, suggesting that these genes did not contribute to salt tolerance in the three lines. Other *HKT* genes were similarly regulated among the three lines. These results indicated that *HKT1*s did not contribute to salt tolerance in these lines and that the *NHX1-like* gene was induced only in salt-tolerant KU-1797 but not in salt-tolerant E58.

### Significant QTLs for SPAD values were not detected in SH wheat

**Figure 6:**
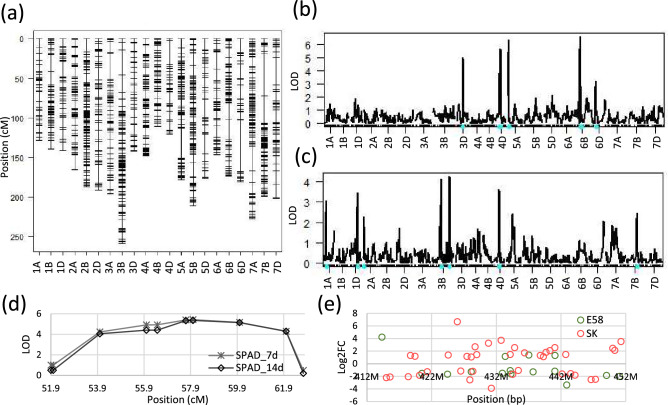
QTL detection revealed by composite interval mapping. (**a**) Genetic map constructed 1330 SNP markers (**b**, **c**) Result of composite interval mapping. The cyan dot indicates the position of QTL. Phenotype data was SPAD value after 7 d of NaCl treatment (**b**) and 14 days of NaCl treatment (**c**). (**d**) Close up of LOD score around QTL on the 4D chromosome. (**e**) Log_2_ transformed fold change of specific DEGs in each line around QTL on the 4D chromosome.

Using an F_2_ population derived from crossing E58 and SK, we attempted to identify QTLs corresponding to DEGs. The F_2_ population contained 91 plants, and their SPAD values at 7 and 14 days after NaCl treatment showed a normal distribution (Fig. 1). We applied an Illumina 90 k Wheat Infinium iSelect SNP array for genotyping. We obtained 4833 SNP markers, which were determined to be genotypes, and successfully determined polymorphisms. These markers were linked and mapped on the IWGSC RefSeq v. 1.0. to confirm the chromosomal position. After deleting duplications, a genetic map for 21 chromosomes was constructed using 1330 markers (Fig. 6). We then performed QTL analysis using these markers by composite interval mapping of SPAD values in leaves after 7 and 14 d of NaCl treatment, respectively (Fig. 6). Six QTLs were detected on 3D, 4D (2 QTLs), 5A, 6B and 6D chromosomes, respectively, based on the SPAD value after 7 d of NaCl treatment. However, there were no significant QTLs because LOD scores were less than 0.05 and 0.1 alpha threshold level, 10.28 and 9.64, respectively. Similarly, seven QTLs were detected on 1A, 1D, 2A, 3B (2 QTLs), 4D, and 7B chromosomes, respectively, for SPAD values after 14 d of NaCl treatment, but their LOD scores were less than 0.05 and 0.1 alpha threshold level, 10.55 and 9.48, respectively. These results suggest that no major QTL contribute to salt tolerance in these wheat lines.

Although statistically significant QTLs were not detected, one QTL on the 4D chromosome was commonly detected in SPAD values at 7 and 14 d after NaCl treatment (Fig. 6). The peak of LOD score was found between two markers, located at 56.5 and 62.7 cM. Referring to the RefSeq genome sequence, we confirmed that their positions were at 409,639,684 bp and 455,253,024 bp of the 4D chromosome of the Chinese Spring genome. Subsequently, we searched for specific DEGs in each line between these two markers, and 51 specific DEGs were found (Fig. 6). *HKT1;4-D* is located on chromosome 4D but is outside the QTL position. It was found that 4 and 11 genes were specifically up-regulated and down-regulated, respectively, in E58 (group a in Fig. 2b,c), and 22 and 16 genes were specifically up-regulated and down-regulated, respectively, in SK (group c and f in Fig. 2b,c). Two genes (TraesCS4D02G258000 and TraesCS4D02G263000) were down-regulated in E58 and up-regulated in SK. The molecular functions characterized by GO annotation were "hydrolase activity" for TraesCS4D02G258000 and "transmembrane transporter activity" for TraesCS4D02G263000. Similarly, four genes up-regulated in E58 were characterized: "nucleic acid binding" for TraesCS4D02G262500, "FAD-binding" for TraesCS4D02G270000, and unknown function for TraesCS4D02G245700 and TraesCS4D02G266400. These might be candidate genes that are partly related to salt tolerance in E58.

## Discussion

Synthetic hexaploid (SH) wheat is likely to have different environmental stress-tolerant responses from common wheat because it contains different gene sets derived from relative wild species. In this study, we used salt-tolerant SH wheat and two common wheat lines, and performed RNA-seq to clarify whether genes related to salt tolerance differed between SH and common wheat. RNA-seq showed that sequence reads from SH and two common wheat lines were almost equally mapped on the RefSeq v. 1.0 assembly^11^, the genome of common wheat cv. Chinese Spring; therefore, genes were characterized on a genome-wide scale (Supplementary Table S1).

To characterize the salt-responsive genes in SH and common wheat, DEGs in response to 6 h of NaCl treatment in roots were identified in each line. The number of DEGs in SH wheat tended to be lower than that in the two common wheat lines, suggesting that the gene expression of common wheat relates more susceptible to salt stress than SH wheat. Our results showed that 9603 DEGs detected in the SH wheat were more than 5128 DEGs identified by transcriptome analysis using a 12 h of NaCl treatment in roots of a salt-tolerant cultivar^14^. Although it cannot be compared with the previous study^14^ because the time of NaCl treatment and definition of DEGs were different, regardless of the strength of salt-stress tolerance, more genes were differentially expressed in common wheat in response to salt stress than those in SH wheat. Additionally, more DEGs were shared between the two common wheat lines than between SH and common wheat (Fig. 2). Hierarchical clustering also showed that the expression levels of the two common wheat lines were correlated and distinguished from SH wheat (Fig. 2). Thus, it is suggested that the expression of many genes is similar in common wheat and is differently regulated in SH wheat in response to salt stress. These DEGs in SH wheat (groups a and e in Fig. 2) were preferentially mapped on the chromosome of the D genome. It was reported that the salt tolerance of SH wheat was derived from D genome donor, *Ae. taucshii*^10^, and newly synthesized wheat showed enhanced salt tolerance by the genes from the D genome^20,23^. Therefore, the acquisition of salt tolerance in SH wheat could be introduced from D-genome wild species with alleles that are different from those of common wheat.

Besides the number of salt-responsive genes, gene functions differed between SH and common wheat, as revealed by GO enrichment analysis (Fig. 3) and KEGG pathway analysis (Table 2). After 6 h of NaCl treatment in roots, genes whose expression was up-regulated were preferentially characterized as phosphorylation and kinase in SH wheat (Fig. 3). Various phosphorylation and kinase signaling pathways work when plants are exposed to abiotic stress^24,25^. One example is the phenylpropanoid biosynthesis pathway, which was enriched in up-regulated genes in SH wheat and down-regulated genes in three lines as shown by KEGG pathway analysis (Table 2). Several kinases are involved in the phenylpropanoid biosynthesis pathway, activated under abiotic stress conditions including salt stress, resulting in the accumulation of various phenolic compounds that scavenge ROS^26^. Although it is still unclear which signaling pathways enhance salt tolerance specific to SH wheat and which signal transduction pathways different from common wheat might be induced in SH wheat.

In contrast, genes with transmembrane transport functions were preferentially up-regulated in the two common wheat lines (Fig. 3). The SOS pathway has been well studied as an important pathway for transporting sodium from the cell^3^. This pathway is conserved in common wheat^19^. SOS1 has transport activity and was more induced by salt stress in salt-tolerant common wheat than in salt-tolerant SH wheat (Fig. 5), indicating that this pathway mainly contributes to salt tolerance in common wheat. We also found a homolog of NHX, expected to have a transport function, showing up-regulation in salt-tolerant common wheat and not in SH wheat (Fig. 5). This gene might also play an important role in salt tolerance in common wheat. These results suggest that up-regulation of transport activities is more crucial for salt tolerance in common wheat than in SH wheat.

The expression of genes related to redox was up-regulated in SH wheat, but was down-regulated in two common wheat lines (Fig. 3). As redox-related genes, the expression of several *RBOH*s, which generate ROS, was up-regulated over time in SH wheat but not in common wheat (Figs. 4 and 5). Among the 10 *RBOH* genes in Arabidopsis, *AtRbohD* and *AtRbohF* are induced in response to salt stress^27^. In addition, a study reported that inducing *NtRbohE* expression enhances salt stress tolerance in tobacco^28^. These results indicate that the *RBOH*s induced by NaCl treatment in SH wheat should enhance salt tolerance in SH wheat.

Transcription factors (TFs), such as NAC, MYB, WRKY, bZIP, and ERF/DREB, regulate abiotic and biotic stress-responsive genes^29^. ERFs belong to the DREB/CBF subfamilies of the plant-specific AP2/ERF TF family^30^. In this study, nine genes annotated with GO terms for transcription activity were up-regulated in SH wheat and down-regulated in common wheat lines (Figs. 4 and 5). They all exhibited homology with ERFs in rice and homoeologous genes named *TaERF-like* on group 5 chromosomes (TraesCS5A02G314600, TraesCS5B02G315500, TraesCS5D02G320800) have high homology with *OsERF68* in rice. A study reported that *OsERF68* is induced by cold stress and seems to contribute to cold tolerance in indica weedy rice but not in japonica cultivars^31^. Although salt stress response is different from the cold stress response, *TaERF-like* might contribute to salt-stress tolerance only in a limited genetic background such as SH wheat used in this study. In contrast, a study showed that *TaERF3* expression is higher when induced by salt stress in salt-tolerant cultivars than in sensitive cultivars, and *TaERF3* overexpression enhanced tolerance to salt and drought in wheat^21^. Similarly, *TaERF3* expression was up-regulated in both salt-tolerant common and SH wheat and not up-regulated in sensitive wheat (Fig. 5). Unlike *TaERF-like* gene, *TaERF3* could promote salt stress tolerance in various genetic backgrounds in wheat.

**Figure 7:**
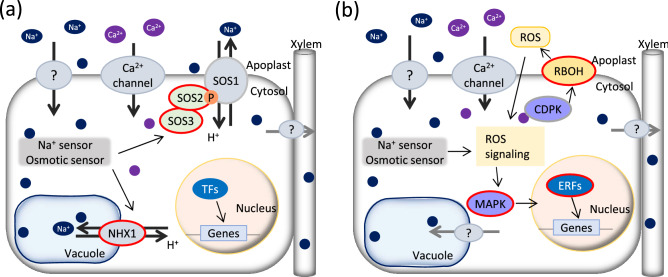
Predicted model of early salt response in common wheat and SH wheat. (**a**) Salt response in common wheat. (**b**) Salt response in SH wheat. Red circles indicate proteins encoded by DEGs whose expression was induced by salt stress.

Collectively, we revealed differentially controlled salt tolerance in SH and common wheat (Fig. 7). Salt tolerance in SH wheat could be controlled by unknown signaling pathways, ROS signaling, including *RBOH*, and different *ERF*s from those in common wheat. Instead, salt tolerance in common wheat used in this study is thought to be enhanced by activating transporters. Salinity tolerance is influenced by various environmental factors^32^, and many QTLs related to salt tolerance have been identified in wheat; for example, 844 QTLs for salinity stress tolerance were collected by meta-QTL analysis^33^. Since DEGs were distributed throughout the chromosome (Figs. S2, S3 and S4) and many genes with negligible contributions were suggested to be involved in salt tolerance in a complex manner, it was considered that no major significant QTL could be detected by one experiment in the F_2_ population. In conclusion, SH wheat has different salt tolerance mechanisms than common wheat (Fig. 7); therefore, SH wheat is expected to benefit future breeding programs for salt tolerance.

## Material and methods

### Plant materials and salt-treatment

Common wheat cv. Shirasagi-komugi (KT020-019: SK) and KU-1797 were provided by the National BioResource Project-Wheat (NBRP) with support by the MEXT, Japan. SK was selected as a salt-tolerant line among Japanese and Chinese landraces conserved by NBRP-Wheat. KU-1797 was screened from the core collection of hexaploid accessions, which was developed by NBRP-Wheat^17^. Synthetic wheat Elite#1–58 (E58) was provided by CIMMYT, Mexico. Experimental research on cultivated varieties were complied with relevant institutional, national, and international guidelines and legislation. Seeds were incubated on a wet paper towel at 4 °C for 3 days under dark conditions. The seeds were transferred and placed at 22 °C for 2 d for uniform germination. The germinated seeds were inserted into the holes in the foam polystyrene floats and floated on 1/5 diluted Murashige-Skoog medium (MS medium, Nihon Pharmaceutical, Tokyo, Japan). Seedlings were grown at 22 °C with a 16 h/8 h (light/dark) for two weeks. The 1/5 diluted MS medium was replaced weekly. Seedlings at three-leaf stage were transferred to a 1/5 diluted MS medium containing 150 mM NaCl, which induced salt stress. The roots were collected at 0, 6, 24, and 72 h after NaCl treatment, frozen immediately in liquid nitrogen, and stored at – 80 °C for RNA extraction. To evaluate the phenotypes, plants were grown for 14 d under the same NaCl treatment, and chlorophyll contents in second leaves were measured using Soil and Plant Analyzer Development (SPAD) chlorophyll meter (SPAD-502, Konica Minolta, Osaka, Japan).

### RNA extraction and sequencing

Total RNA was extracted using the RNeasy Plant Mini Kit (QIAGEN, Hilden, Germany) according to the manufacturer’s instructions and treated with DNase I (Takara Bio, Otsu, Japan). The quality and concentration of the RNA were checked using a NanoDrop 1000 (ND-1000, Thermo Fisher Scientific, MA, USA) and Qubit assay (Qubit, Thermo Fisher Scientific, MA, USA). RNAs from roots at 0 and 6 h after NaCl treatment were used for RNA sequencing because several DEGs have been observed in roots after 6 h of 150 mM NaCl treatment^34^. Sequencing was performed in three biological replicates. Library construction and paired-end sequencing on a HiSeq 2500 (Illumina, San Diego, CA, USA) were conducted by Eurofins Genomics (Tokyo, Japan) and GENEWIZ Japan (Tokyo, Japan).

### Expression analysis and GO enrichment analysis

The quality of the row reads was checked using FastQC ver. 0.11.5 (https://www.bioinformatics.babraham.ac.uk/projects/fastqc/) and trimmed with Trimmomatic ver. 0.36^35^. The reads were then aligned to RefSeq v. 1.0 released by IWGSC using HISAT2 ver. 2.1.0^36^ with default parameters. The alignments were sorted using the SAMtools ver. 1.6^37^ and converted to the BAM format. Reads were counted using featureCounts in the rsubread R^38^ package ver. 1.6.0, and differentially expressed genes (DEGs) were analyzed using DESeq2^39^. Genes with a P-value of less than 0.01 and an absolute log_2_ value of fold change (Log2FC) of more than 1 were determined to be significant DEGs. DEGs were characterized by GO enrichment analysis using the singular enrichment analysis tool on agriGO ver. 2.0^40^ with default parameters. The GO enrichment score was converted into the absolute value of log_10_ for each GO term.

### KEGG pathway analysis

Sequences of cDNA in wheat were obtained from EnsemblPlants (http://plants.ensembl.org/) and used as queries. In addition, a BLASTX search was conducted to rice amino acid sequence from RAP-DB (https://rapdb.dna.affrc.go.jp/). Top-hit genes which have an E-value less than 1.0E-4 were determined as orthologs. These rice genes were used for KEGG^18^ pathway enrichment analysis by CARMO^41^.

### Expression analysis using qRT-PCR

Total RNA was treated with recombinant DNase I (Takara Bio, Japan) and 1 μg RNA samples were used to synthesize cDNA using oligo(dT)_20_ primer and ReverTra Ace (Toyobo, Japan). In addition, gene expression level was measured using SYBR Premix Ex Taq II (Takara Bio, Japan) on a Thermal Cycler Dice Takara Dice Real-Time System TP800 (Takara Bio). Primers, designed using Primer3^42^, are listed in Supplementary Table S2. Gene expression level relative to *Actin* expression level was calculated using the ΔCT method.

### QTL analysis

Synthetic wheat E58 and SK were crossed to produce the F_2_ population. Genomic DNA was extracted from each plant after phenotyping. Genotyping of each plant was performed using a 90 k Wheat Infinium iSelect SNP array^43^. A linkage map was constructed using the OneMap package in R software^44^. QTL mapping was performed using composite interval mapping with the cim function^45^ in R/qtl package^46^. The calculation used Haley and Knott regression method and set the window size to 10 cM and the number of covariates to 7. The threshold’s 0.05 and 0.1 alpha level was determined from 1000 permutations.

## Supplementary Information

Supplementary Tables.

Supplementary Figures.

## Data Availability

The raw reads generated during the current study are available in the DDBJ sequence read archive, DRR346657–DRR346674. The datasets analyzed during the study are available in the DDBJ Genomic Expression Archive (GEA), E-GEAD-479.
