# Supplementary material for: Comparative transcriptome analysis of synthetic and common wheat in response to salt stress
Source: Sci Rep. 2022 Jul 7;12:11534. doi: 10.1038/s41598-022-15733-2 (PMC9262916; doi:10.1038/s41598-022-15733-2)
Supplement: Supplementary file 1 — Supplementary Tables. [file 41598_2022_15733_MOESM1_ESM.docx]

Supplemental Table S1 Summary of RNA-seq reads and assembly statics

|  | Treat | Replicate | No. reads | Yield  (Gb) | Q30  (%) | No. cleaned reads | No. reads uniquely aligned | % |
| --- | --- | --- | --- | --- | --- | --- | --- | --- |
| KT020-019 | Control | R1 | 45,931,162 | 4.593 | 94.7 | 21,476,645 | 17,027,486 | 79.3 |
| (SK) |  | R2 | 21,934,180 | 6.580 | 92.8 | 15,412,102 | 12,012,574 | 77.9 |
|  |  | R3 | 21,928,401 | 6.579 | 93.2 | 16,066,881 | 12,570,565 | 78.2 |
|  | Salt | R1 | 31,701,348 | 3.170 | 94.4 | 14,794,761 | 12,515,947 | 84.6 |
|  |  | R2 | 44,443,478 | 13.33 | 92.6 | 33,313,547 | 27,517,971 | 82.6 |
|  |  | R3 | 23,137,191 | 6.941 | 92.2 | 16,296,691 | 11,641,657 | 71.4 |
| Elite#1-58 | Control | R1 | 36,584,216 | 3.658 | 96.3 | 17,572,616 | 14,606,130 | 83.1 |
| (E58) |  | R2 | 24,624,427 | 7.387 | 92.8 | 18,372,601 | 14,523,713 | 79.1 |
|  |  | R3 | 28,108,915 | 8.433 | 92.5 | 20,641,393 | 16,021,747 | 77.6 |
|  | Salt | R1 | 31,835,706 | 3.183 | 96.1 | 15,305,672 | 12,788,447 | 83.6 |
|  |  | R2 | 34,659,758 | 10.40 | 92.5 | 25,850,964 | 20,147,503 | 77.9 |
|  |  | R3 | 27,831,354 | 8.349 | 92.2 | 20,751,960 | 16,024,036 | 77.2 |
| KU-1797 | Control | R1 | 34,104,326 | 3.410 | 96.1 | 16,310,435 | 13,629,126 | 83.6 |
|  |  | R2 | 38,330,932 | 3.833 | 96.2 | 18,304,654 | 15,444,055 | 84.4 |
|  |  | R3 | 29,178,730 | 8.754 | 92.8 | 21,431,984 | 14,561,187 | 67.9 |
|  | Salt | R1 | 37,514,884 | 3.751 | 96.1 | 18,037,779 | 15,399,367 | 85.4 |
|  |  | R2 | 34,682,456 | 3.468 | 96.2 | 16,664,934 | 12,573,318 | 75.4 |
|  |  | R3 | 26,988,963 | 8.097 | 92.3 | 20,076,606 | 16,319,053 | 81.3 |

Supplemental Table S2 List of primers used in qRT-PCR

| Primer name | Sequence (5' -> 3') | Target gene | Reference |
| --- | --- | --- | --- |
| Actin-A | CACTGGAATGGTCAAGGCTG | TraesCS1A02G274400, TraesCS1B02G283900, TraesCS1D02G274400 | Rong et al. (2014) |
| Actin-B | CTCCATGTCATCCCAGTTG |  |  |
| TaSOS1_3058F | GAGCATGTCGGTTTGCTCAGG | TraesCS3A02G023200, TraesCS3D02G022900 |  |
| TaSOS1_3198R | CGTCATGCTGCCATACATGC |  |  |
| TaSOS2_913F | GGTGGTCCTCTTATGATGAATGC | TraesCS7B02G279300 |  |
| TaSOS2_1032R | CCTTGATACGAAGCGAGTTTGG |  |  |
| TaSOS3_723F | ATTCAAGCAGGCAGACCTGAAC | TraesCS1A02G261200, TraesCS1B02G272000, TraesCS1D02G261200 |  |
| TaSOS3_876R | GACAACAAAGCTGGGGAATGAC |  |  |
| TaSOS3_746F | ACAGGATAGACCCCAAGGAGTG | TraesCS1D02G358400 |  |
| TaSOS3_883R | TGTAATCGTCGACTTCCGAATG |  |  |
| TaSOS1_2971F | GAAGTGCATCAATGCTGTCTCC | TraesCS7B02G475500 |  |
| TaSOS1_3117R | TACCGGGTTGGTTTTTGATTTC |  |  |
| NXH1L_2501F | ACAGCAAGAGAAGGAAGCGAAG | TraesCS2B02G141900 |  |
| NXH1L_2647R | CGCATCACCGTTACTAGGGTTC |  |  |
| TaERFL_608F | GCTCCTGCGAGGAGGTGAAG | TraesCS5A02G314600, TraesCS5B02G315500, TraesCS5D02G320800 |  |
| TaERFL_688R | TGTAGGGGACCCCGAGGAAG |  |  |
| TaERF3-QF | AGCAATCAGGCAAAGCAACC | TraesCS2D02G414600 | Rong et al. (2014) |
| TaERF3-QR | ACGACTCAGAAGGAACCACGAC |  |  |
| RBOH-F | TTCTACTGCGGCGACCAGAAG | TraesCS5A02G093600, TraesCS5B02G099700, TraesCS5D02G105900 |  |
| RBOH-R | AGACGAACTTGGTGGTGGTCC |  |  |
