# Supplementary material for: Comparative transcriptome analysis of synthetic and common wheat in response to salt stress
Source: Sci Rep. 2022 Jul 7;12:11534. doi: 10.1038/s41598-022-15733-2 (PMC9262916; doi:10.1038/s41598-022-15733-2)
Supplement: Supplementary file 2 — Supplementary Figures. [file 41598_2022_15733_MOESM2_ESM.pdf]

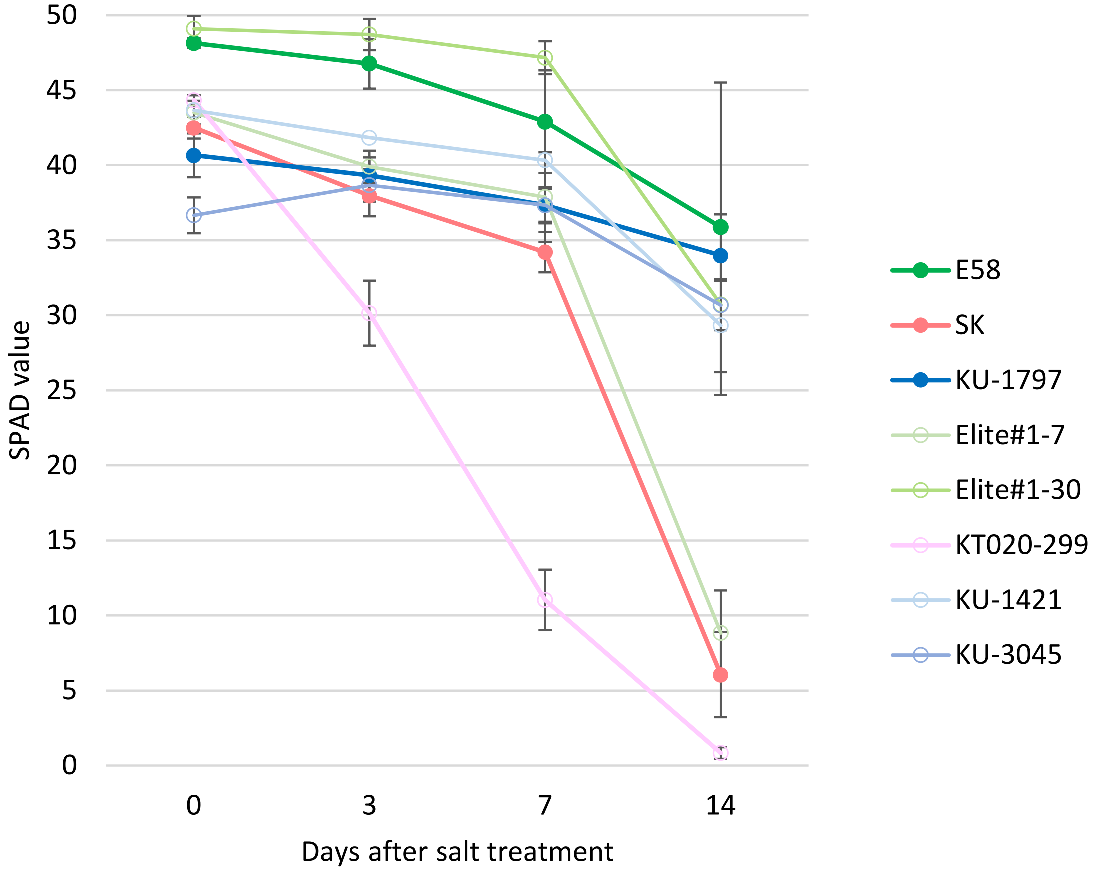

Supplementary Figure 1 SPAD value of leaf after 150 mM NaCl treatment.

E58 (Elite#1-58), Elite#1-7 and Elite#1-30 were provided by CIMMYT. SK (KT020-019), KT020-299, KU-1797, KU-1421 and KU-3045 were provided by the National BioResource Project-Wheat. Error bar shows standard error.

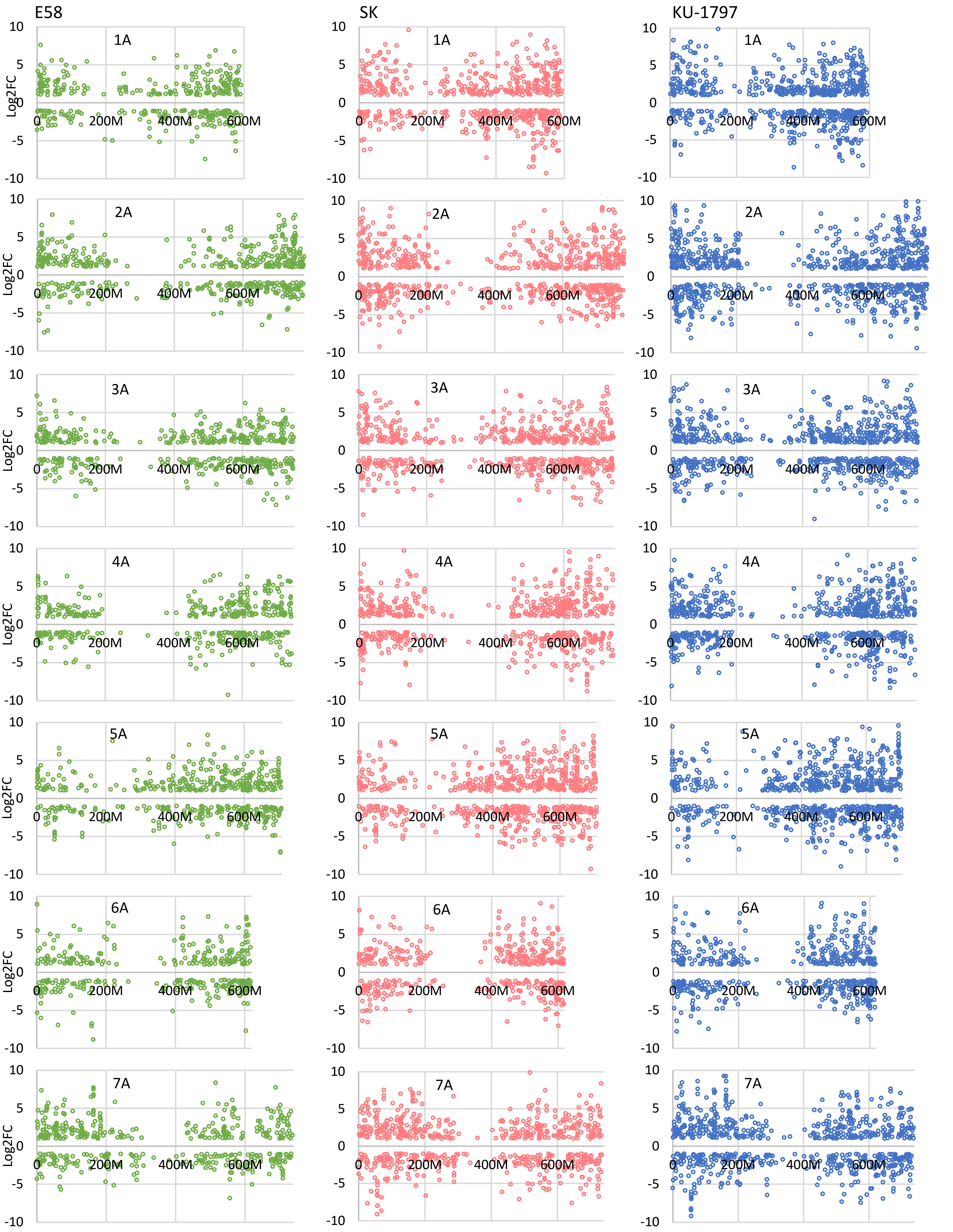

Supplementary Figure 2 DEGs mapped to A genome chromosomes in E58, SK and KU-1797 and their log2 converted fold changes (Log2FC).

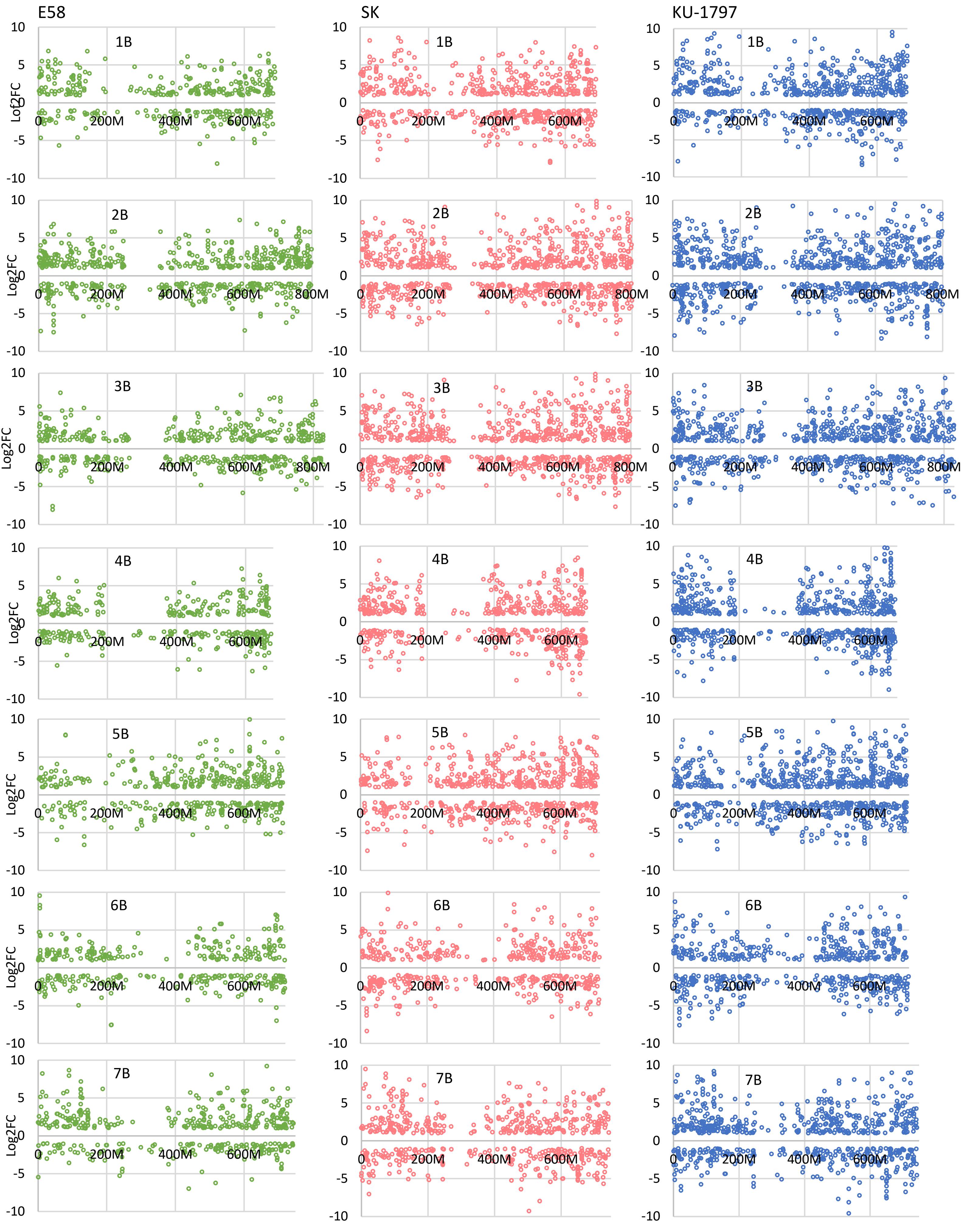

Supplementary Figure 3 DEGs mapped to B genome chromosomes in E58, SK and KU-1797 and their log2 converted fold changes (Log2FC).

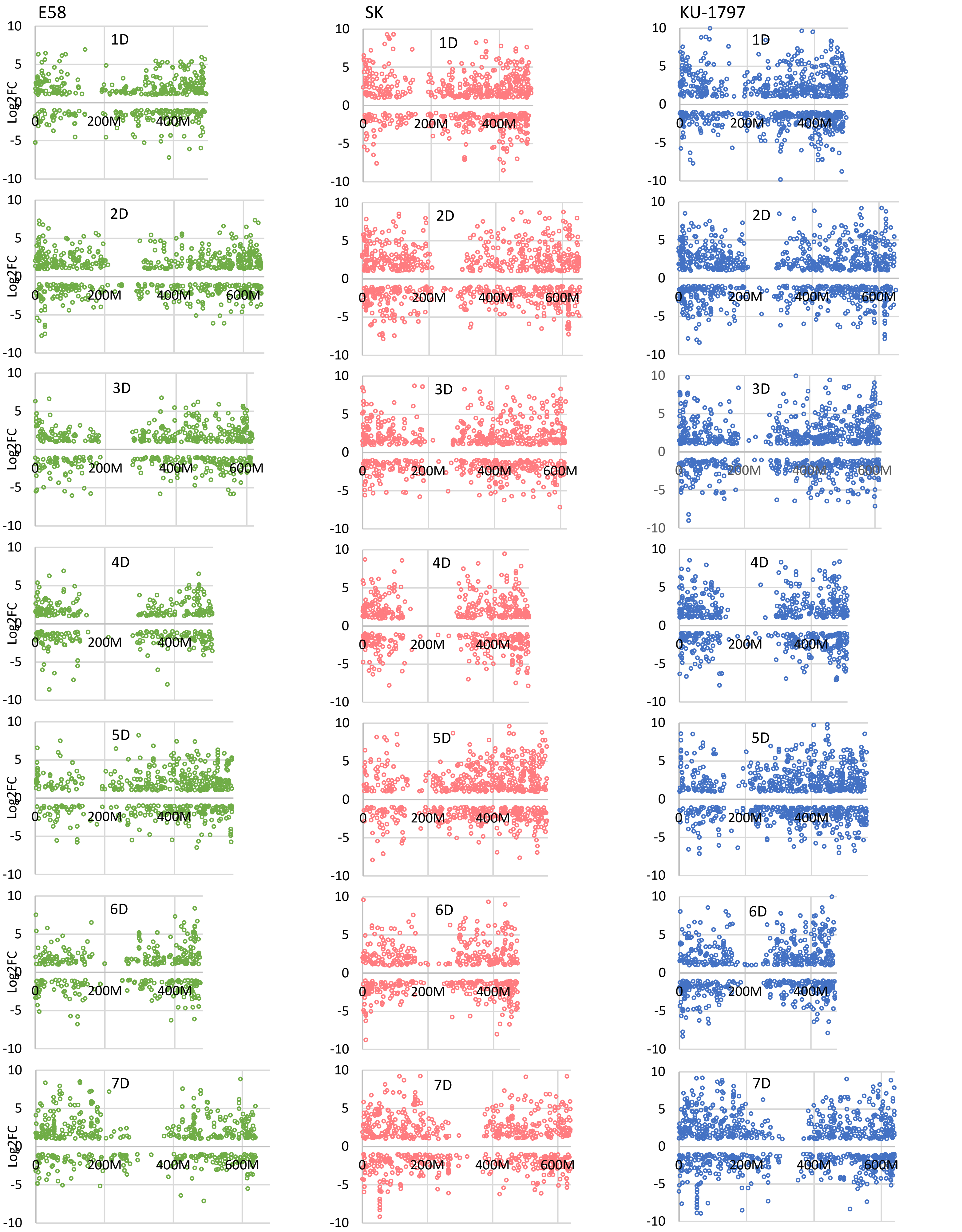

Supplementary Figure 4 DEGs mapped to D genome chromosomes in E58, SK and KU-1797 and their log2 converted fold changes (Log2FC).

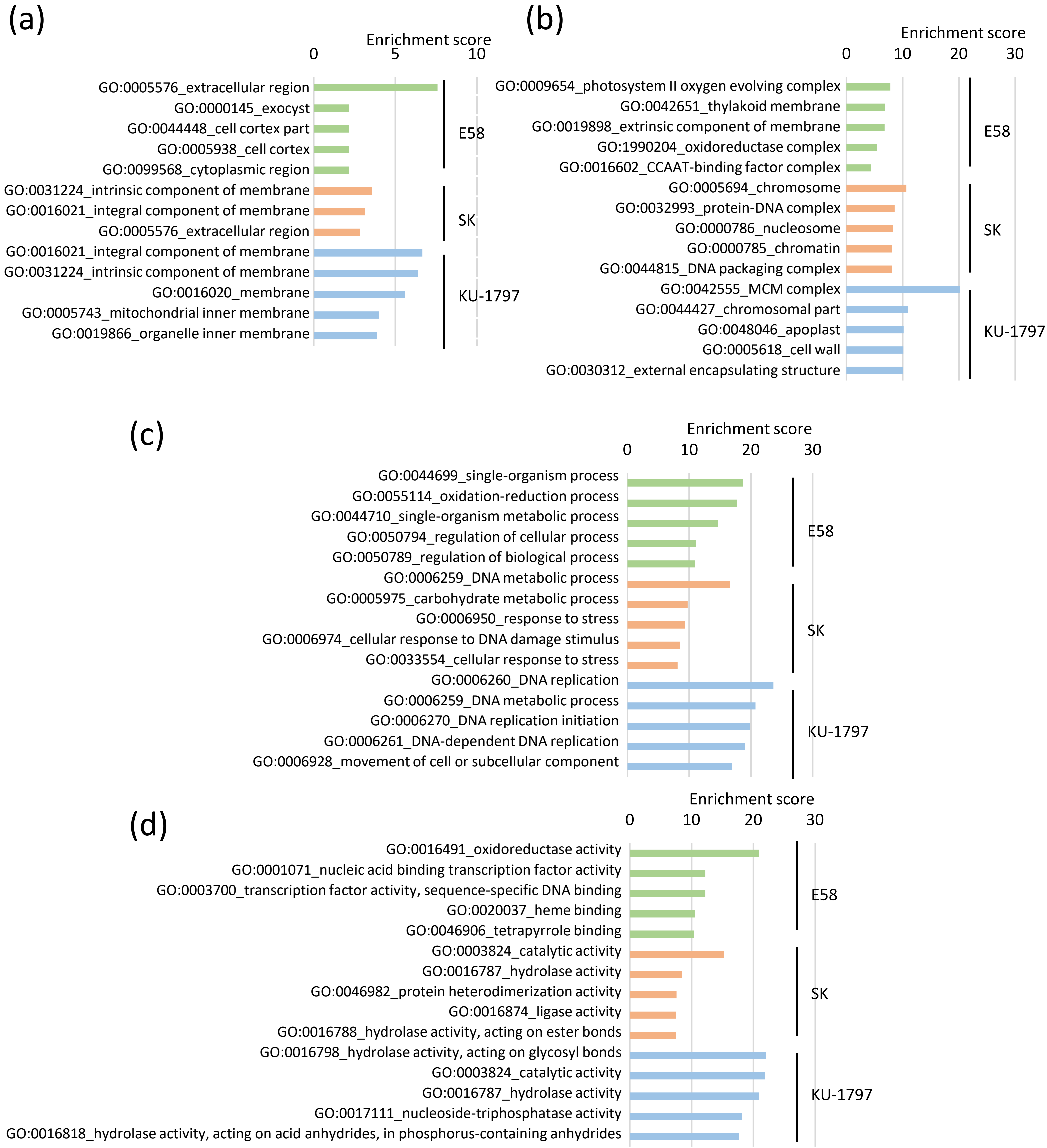

Supplementary Figure 5 GO enrichment analysis for specific DEGs in each line
(a) Cellular component enriched in each line specific up-regulated DEGs (b) Cellular component enriched in each line specific down-regulated DEGs (c) Biological process enriched in each line specific down-regulated DEGs (d) Molecular function enriched in each line specific down-regulated DEGs
